# Concurrent Anti-glomerular Basement Membrane (Anti-GBM) Disease and Immunoglobulin A Nephropathy (IgAN) Presenting With Severe Pulmonary-Renal Syndrome: A Case Report

**DOI:** 10.7759/cureus.113164

**Published:** 2026-07-22

**Authors:** Fahd Alshuweishi, Mohammed Almuhraj, Naif J Benragosh, Areej Alqunaitir, Khaled O Alsaad

**Affiliations:** 1 Department of Nephrology, King Saud Medical City, Riyadh, SAU; 2 Department of Internal Medicine, King Saud Medical City, Riyadh, SAU; 3 Department of Anatomical Pathology, King Faisal Specialist Hospital and Research Centre, Riyadh, SAU; 4 Department of Pathology, King Saud Medical City, Riyadh, SAU

**Keywords:** anti-glomerular basement membrane disease, autoimmune glomerulonephritis, crescentic glomerulonephritis, immunoglobulin a nephropathy, pulmonary-renal syndrome, renal biopsy

## Abstract

Anti-glomerular basement membrane (anti-GBM) disease is a rare, aggressive autoimmune condition that typically presents as rapidly progressive glomerulonephritis and can be complicated by pulmonary haemorrhage. The co-occurrence of anti-GBM disease and IgA nephropathy (IgAN) is extremely uncommon, though increasingly recognised as a distinct clinical and pathological entity. We describe a 51-year-old Sudanese man who presented with acute hypoxaemic respiratory failure, haemoptysis, severe anaemia, and acute kidney injury. Testing showed high levels of anti-GBM antibodies, with negative anti-neutrophil cytoplasmic antibodies. Chest imaging revealed diffuse alveolar haemorrhage. Renal biopsy demonstrated crescentic glomerulonephritis with linear IgG staining along the glomerular basement membrane, alongside mesangial deposits dominated by IgA, confirming the presence of both diseases. Treatment included plasma exchange, high-dose corticosteroids, cyclophosphamide, and bronchial artery embolisation, which led to significant respiratory improvement and stabilisation of kidney function. A review of published cases suggests that individuals with this overlap may experience better renal outcomes than those with anti-GBM disease alone, though serious pulmonary-renal symptoms can still occur. This case highlights the need for accurate clinicopathological assessment and prompt initiation of anti-GBM-specific treatment.

## Introduction

Anti‑glomerular basement membrane (anti‑GBM) disease is a rare but fulminant autoimmune disorder characterised by autoantibodies directed against the non‑collagenous domain of the α3 chain of type IV collagen, targeting the glomerular and sometimes alveolar basement membranes [1]. Clinically, it often presents as rapidly progressive glomerulonephritis, frequently accompanied by pulmonary haemorrhage, and remains one of the most aggressive forms of glomerulonephritis despite improvements in therapy [2]. In contrast, immunoglobulin A nephropathy (IgAN), also known as Berger’s disease, is the most common glomerulonephritis worldwide. It is defined by dominant IgA deposition, mainly in the glomerular mesangium, and exhibits a wide clinical spectrum ranging from occult haematuria with a benign course to progressive chronic kidney disease over decades [3,4].

The concurrent occurrence of anti‑GBM disease and IgAN is exceedingly rare. Since the first descriptions in the late 1990s, only a limited number of cases have been reported; two recent comprehensive reviews identified 39 and 61 such patients, respectively [5,6]. These dual glomerulopathies are remarkable not only for their rarity, but also because emerging evidence suggests they may differ phenotypically and prognostically from isolated anti‑GBM disease. Indeed, patients with coexisting anti‑GBM disease and IgAN tend to present with less severe renal impairment, lower rates of oliguria or anuria, and overall better renal outcomes compared to classical anti‑GBM presentations [7,8]. Shen and colleagues found that mesangial IgA deposition was independently associated with protection against progression to end‑stage renal disease (ESRD) in anti‑GBM patients, even when adjusting for hypertension, oliguria, and percentage of crescents [9]. Similarly, Chen et al. noted a milder clinical course and more indolent progression in cases of dual pathology [5].

The pathogenesis underlying this rare overlap remains incompletely understood. One hypothesis proposes that IgA‑mediated mesangial injury may alter the structural conformation of type IV collagen in the glomerular basement membrane, thereby exposing cryptic epitopes and triggering anti‑GBM antibody formation [6,10]. Another theory is that galactose‑deficient IgA1 may form immune complexes that facilitate such autoimmune responses [8,11]. Alternatively, in some patients, IgAN may precede or predispose to anti‑GBM disease through immune complex-mediated inflammation. However, it remains uncertain whether the anti‑GBM disease is primary or secondary to pre‑existing IgAN [5].

Although recognition of this dual entity has increased in recent years, the available literature remains sparse, consisting predominantly of individual case reports and small case series. In this report, we describe a new case of concurrent anti-GBM disease and IgAN, thereby contributing an additional observation to the limited body of evidence. By doing so, we aim to enrich current understanding of this rare overlap and provide further context for clinicians and researchers encountering similar diagnostic and therapeutic challenges. The accompanying literature review is narrative in nature and was based on relevant English-language publications identified through PubMed, with additional studies identified by reviewing the reference lists of retrieved articles.

## Case presentation

A 51-year-old man from Sudan, with no known past medical or surgical history, came to the hospital after experiencing a week-long progression of shortness of breath and a productive cough, which eventually included blood-tinged sputum. On the day of admission, his symptoms suddenly worsened. He developed marked shortness of breath and a noticeable episode of haemoptysis, which was estimated at under 10 mL. A month prior, he had a sore throat that was treated with antibiotics. He also reported losing around 4 kg unintentionally, experiencing occasional low-grade fevers, and intermittently using tobacco and Tambak (a smokeless tobacco product), although he had quit smoking two years ago. There was no personal or family history of autoimmune or kidney-related disease.

Upon admission, he was in hypoxaemic respiratory failure, which required immediate intubation and mechanical ventilation. His vital signs were as follows: blood pressure was 148/85 mmHg, heart rate 114 beats per minute, respiratory rate 30 breaths per minute, temperature 38.2°C, and oxygen saturation 80% on room air. On physical exam, he had bilateral crackles on chest auscultation, and fundoscopy revealed retinal haemorrhages in both eyes.

Initial laboratory work showed signs of acute kidney injury, with a serum creatinine of 259.6 µmol/L (normal: 63.6-110.5 µmol/L) and blood urea nitrogen of 13.9 mmol/L (normal: 3-9.2 mmol/L). His blood counts revealed severe anaemia: haemoglobin was 4.2 g/dL, haematocrit 14.9%, and red blood cell count 2.2 ×10⁶/µL, along with a high red cell distribution width. The white blood cell count was elevated at 14.1 ×10³/µL, mainly neutrophils, and platelets were mildly raised at 400 ×10³/µL. C-reactive protein was significantly increased at 44.5 mg/L, indicating systemic inflammation. Other biochemical findings included hypoalbuminaemia (31 g/L), mild hyponatraemia (135 mmol/L), low calcium (1.84 mmol/L), and a slightly elevated glucose level (8.6 mmol/L). Urine testing showed proteinuria with 368 mg of protein over 24 hours, a urine albumin-to-creatinine ratio of 2791.6 mg/g, and low creatinine levels in the urine.

Serological testing revealed a strong positive for anti-GBM antibodies. Testing for anti-myeloperoxidase (MPO) and anti-proteinase 3 (PR3) antibodies returned negative. Complement levels were within normal ranges (C3 1.03 g/L, C4 0.26 g/L). Screening for hepatitis B, hepatitis C, and human immunodeficiency virus (HIV) was negative. Bone metabolism tests identified a vitamin D deficiency (25-OH vitamin D at 30.4 nmol/L) and a raised parathyroid hormone level (137 ng/L), consistent with secondary hyperparathyroidism. The patient's initial laboratory investigations are summarised in Table 1.

**Table 1:** Initial Laboratory Investigations at Presentation

Laboratory test	Patient result	Reference range
Haematology		
Haemoglobin	4.2 g/dL	13.0-17.0 g/dL
Haematocrit	14.9%	40-50%
Red blood cell count	2.2 ×10⁶/µL	4.5-5.9 ×10⁶/µL
Red cell distribution width	Increased (numerical value unavailable)	11.5-14.5%
White blood cell count	14.1 ×10³/µL	4.0-11.0 ×10³/µL
Platelet count	400 ×10³/µL	150-400 ×10³/µL
Renal function and biochemistry		
Serum creatinine	259.6 µmol/L	63.6-110.5 µmol/L
Blood urea nitrogen	13.9 mmol/L	3.0-9.2 mmol/L
C-reactive protein	44.5 mg/L	<5 mg/L
Albumin	31 g/L	35-50 g/L
Sodium	135 mmol/L	136-145 mmol/L
Calcium	1.84 mmol/L	2.15-2.55 mmol/L
Glucose	8.6 mmol/L	3.9-7.8 mmol/L
Urine studies		
24-hour urine protein	368 mg/24 h	<150 mg/24 h
Urine albumin-to-creatinine ratio	2791.6 mg/g	<30 mg/g
Urine creatinine	Decreased (value unavailable)	Laboratory dependent
Serology and immunology		
Anti-glomerular basement membrane (anti-GBM) antibody	Positive	Negative
Myeloperoxidase anti-neutrophil cytoplasmic antibody (MPO-ANCA)	Negative	Negative
Proteinase 3 anti-neutrophil cytoplasmic antibody (PR3-ANCA)	Negative	Negative
Complement component 3 (C3)	1.03 g/L	0.90-1.80 g/L
Complement component 4 (C4)	0.26 g/L	0.10-0.40 g/L
Hepatitis B serology	Negative	Negative
Hepatitis C serology	Negative	Negative
Human immunodeficiency virus (HIV) serology	Negative	Negative
Bone metabolism		
25-hydroxyvitamin D	30.4 nmol/L	>50 nmol/L
Parathyroid hormone	137 ng/L	15-65 ng/L

**Figure 1:**
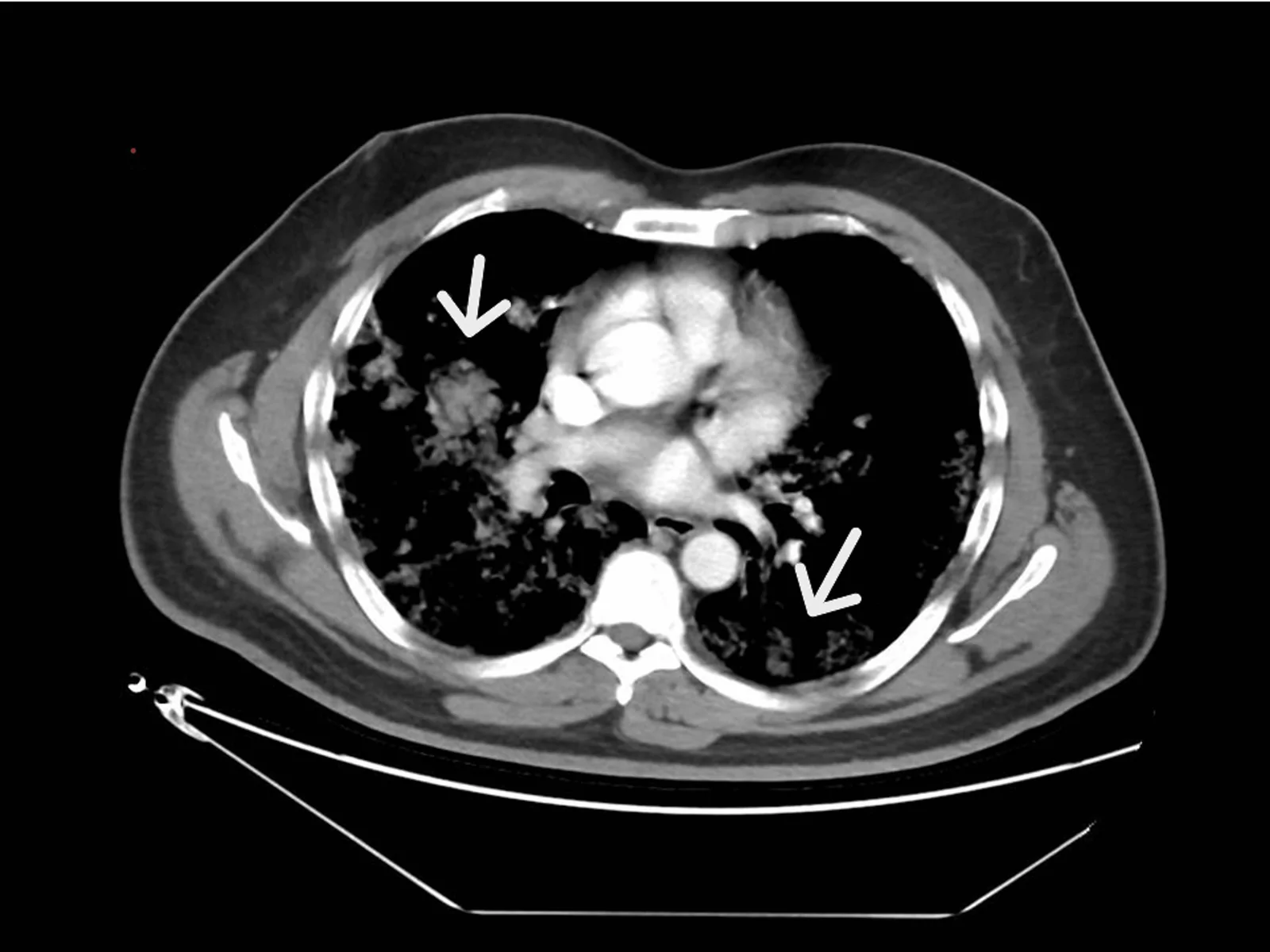
Axial Chest Computed Tomography of the Chest Axial chest computed tomography of the chest demonstrating bilateral multifocal air-space opacities, including patchy consolidative changes (arrows) and surrounding ground-glass attenuation, in keeping with diffuse alveolar haemorrhage in the clinical context of anti-glomerular basement membrane disease.

A high-resolution computed tomography (CT) scan of the chest showed diffuse ground-glass opacities and multifocal patchy areas of consolidation in both lungs (Figure 1). These findings strongly supported a diagnosis of diffuse alveolar haemorrhage rather than a thromboembolic process. Renal ultrasound showed both kidneys were normal in size, but there was increased cortical echogenicity.

**Figure 2:**
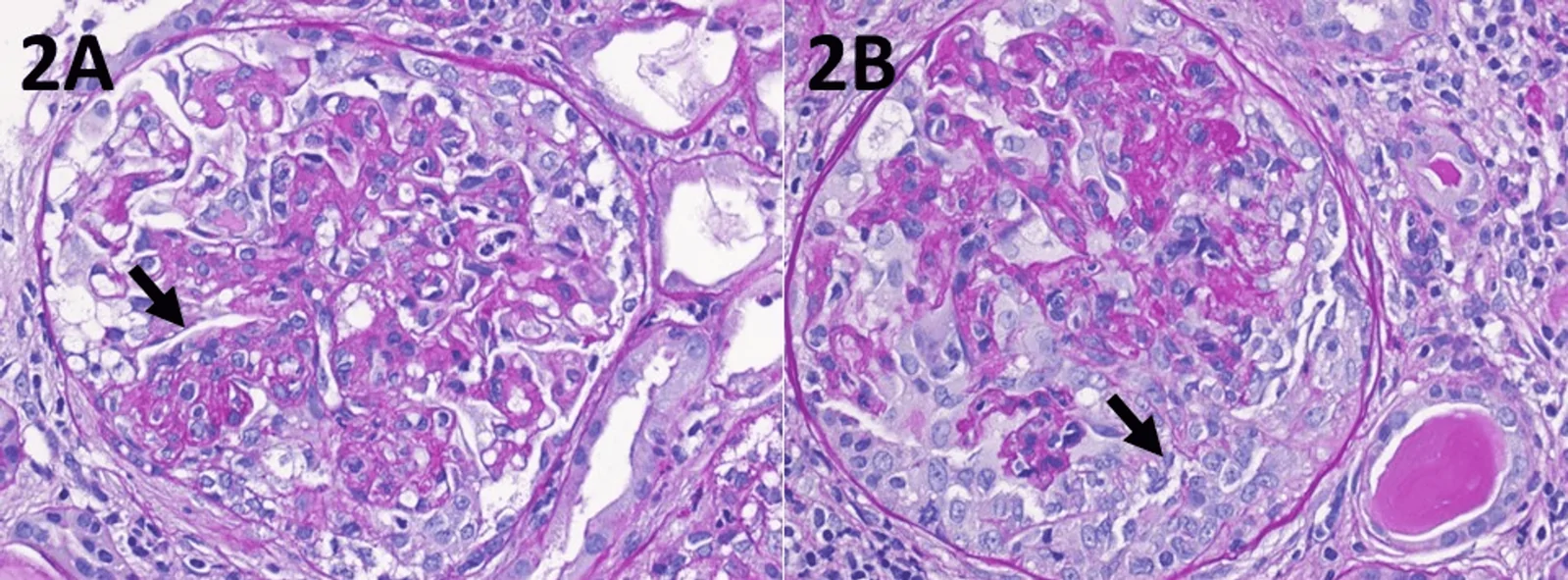
Light Microscopy (A) Glomerulus showing mild to moderate mesangial expansion with increased mesangial matrix and cellularity (arrow). (B) Glomerulus demonstrating a cellular crescent composed mainly of proliferating epithelial cells, consistent with crescentic glomerulonephritis (arrow) (Periodic acid-Schiff stain; original magnification x400).

A percutaneous biopsy of the left kidney was performed and yielded 11 glomeruli, with one showing global sclerosis. On light microscopy, there was mild to moderate expansion of the mesangial matrix and mesangial hypercellularity (Figure 2A). Segmental scarring was seen in three glomeruli, and crescents were found in five; four cellular and one fibrocellular (Figure 2B). No endocapillary hypercellularity, subendothelial deposits, significant karyorrhectic debris, fibrinoid necrosis, or microthrombi were identified. The tubulointerstitial compartment showed moderate acute tubular injury, mild chronic lymphocytic inflammation with scattered plasma cells, and focal mild interstitial fibrosis and tubular atrophy. Mild sclerosis of small arteries was also observed.

**Figure 3:**
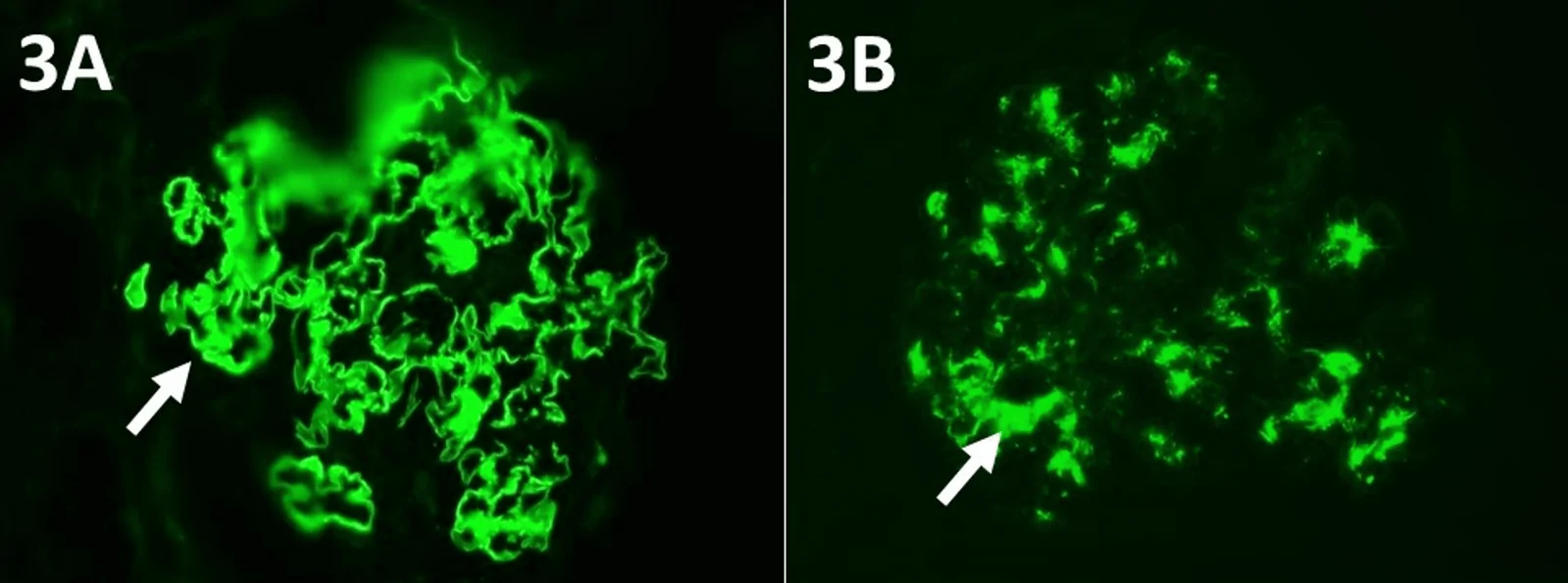
Immunofluorescence Microscopy (A) Immunofluorescence microscopy showing strong, diffuse linear IgG staining along the glomerular basement membranes, diagnostic of anti-GBM disease (arrow). (B) Immunofluorescence microscopy demonstrating granular mesangial IgA-dominant staining, consistent with concurrent IgAN (arrow). IgAN: immunoglobulin A nephropathy; IgG: immunoglobulin G; anti-GBM: anti-glomerular basement membrane

**Figure 4:**
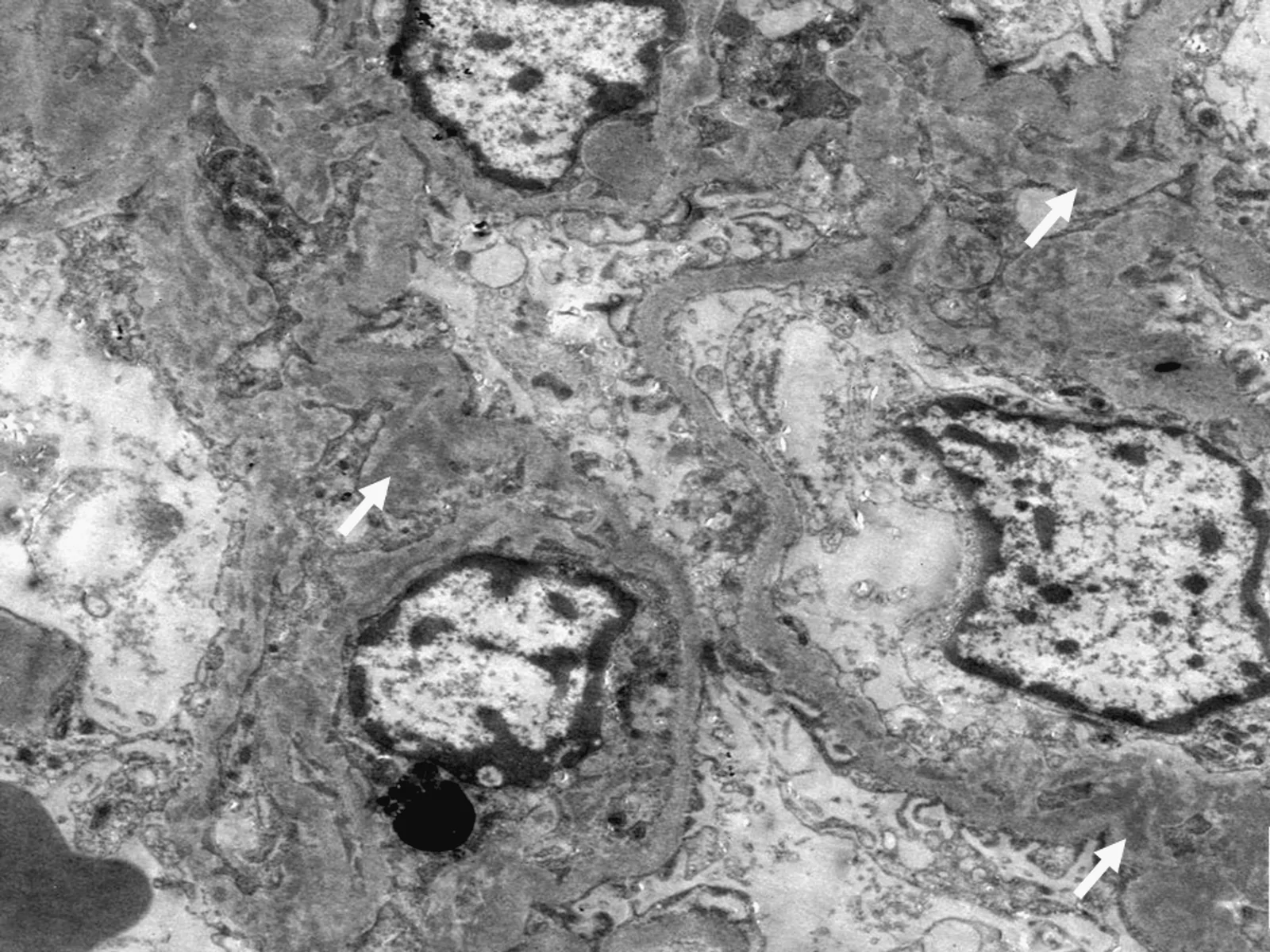
Electron Microscopy Electron microscopy demonstrating mesangial and paramesangial immune-type electron-dense deposits (arrows), supporting the diagnosis of concurrent IgAN (electron microscopy; uranyl acetate and lead citrate stain; original magnification ×1,000). IgAN: immunoglobulin A nephropathy

Immunofluorescence microscopy showed strong, diffuse linear staining of the glomerular capillary walls for IgG (3+) (Figure 3A), along with kappa (3+) and lambda (2+) light chains, consistent with anti-GBM disease. There was also fine and coarse granular mesangial staining for IgA (2+) (Figure 3B), weaker staining for IgM (1+), C3 (1+), and trace positivity for C1q, indicating coexistent IgAN. On electron microscopy, mesangial and paramesangial immune-type electron-dense deposits were found, with mild expansion of the mesangial matrix (Figure 4). The glomerular basement membranes showed mild segmental thickening and segmental foot process effacement, but no double contours, attenuation, or lamellation were seen. No deposition of organised structures was identified.

Taken together, the clinical presentation, serological findings, and kidney biopsy results supported a diagnosis of crescentic glomerulonephritis due to anti-GBM disease, occurring alongside IgAN. The patient underwent bronchial artery embolisation to manage the haemoptysis and received seven sessions of plasma exchange. He was started on oral cyclophosphamide at 2 mg/kg/day for three months and prednisone at a dose of 40 mg/day. Concurrent antibiotics, doxycycline and piperacillin-tazobactam, were also given.

Over the hospital stay, his breathing improved considerably, and he was successfully taken off the ventilator on the sixth day. Although his kidney function did not return to normal, it remained stable. He was discharged home on a tapering course of corticosteroids and scheduled for close follow-up with nephrology.

A summary of the key clinical features, histopathological characteristics, treatment, and short-term outcome is provided in Table 2.

**Table 2:** Summary of Key Clinical Features, Pathology, Treatment, and Outcome IgA: immunoglobulin A; IgG: immunoglobulin G; IgM: immunoglobulin M

Domain	Findings
Demographics	51-year-old Sudanese male, ex-smoker, occasional Tambak use
Presentation	Progressive dyspnoea, cough with haemoptysis, weight loss, fever
Examination	Respiratory failure requiring intubation, tachycardia, fever, retinal haemorrhages
Imaging	Computed tomography chest: diffuse ground-glass opacities and consolidations (alveolar haemorrhage)
Biopsy	Light microscopy: 11 glomeruli; five crescents; segmental sclerosis; Immunofluorescence: linear IgG (3+) along glomerular basement membranes; mesangial IgA (2+), IgM, complement 3 (C3); Electron microscopy: mesangial/paramesangial deposits
Treatment	Bronchial artery embolisation, seven plasma exchanges, cyclophosphamide, prednisone, antibiotics
Outcome	Extubated by day 6, respiratory improvement, renal impairment persisted but stable; discharged on steroid taper with nephrology follow-up

## Discussion

Anti-GBM disease is an uncommon but highly aggressive autoimmune condition. It is driven by circulating IgG autoantibodies that target the non-collagenous (NC1) domain of the α3 chain of type IV collagen found in the glomerular and alveolar basement membranes. Most commonly, it presents as rapidly progressive crescentic glomerulonephritis, often accompanied by diffuse alveolar haemorrhage, together forming the classic pulmonary-renal syndrome. Despite improvements in treatment with immunosuppressive agents and plasma exchange, outcomes are still closely tied to the severity of disease at diagnosis. In particular, patients presenting with advanced kidney failure, dialysis dependence, or widespread crescentic involvement generally have poor renal outcomes [1,12,13].

In contrast, IgAN is the most widespread primary glomerulonephritis globally. It is marked by dominant or co-dominant mesangial IgA deposition on kidney biopsy. The clinical course is variable, ranging from isolated microscopic haematuria to slow, progressive chronic kidney disease or, less commonly, crescentic glomerulonephritis [14]. Although the coexistence of anti-GBM disease and IgAN is rare, it is now more often recognised as a distinct clinicopathological overlap rather than a coincidental occurrence of two unrelated diseases [5,6,8,9,15].

Over the last decade, accumulating evidence from case reports, small series, and systematic reviews has improved our understanding of this overlap. More recent analyses suggest that patients with both anti-GBM disease and IgAN often experience better renal outcomes compared to those with anti-GBM disease alone [5,6,9,15]. Specifically, these patients are more likely to present with lower serum creatinine, maintained urine output, and a much lower incidence of oliguria or anuria, features that are strongly associated with improved renal prognosis in anti-GBM disease [5,6,9]. Importantly, mesangial IgA deposition has emerged as an independent protective factor against progression to ESRD, even after adjusting for key clinical and histological variables such as baseline renal function, crescent burden, and treatment approach [5]. These findings are further supported by published case reports, including that of Shaojie et al. [7], which describe substantial renal recovery following timely initiation of standard anti-GBM therapy in patients with concurrent IgAN. Together, these findings suggest that IgA-driven immune processes may influence the severity or progression of anti-GBM-related injury. However, the available evidence remains limited and is derived predominantly from individual case reports, small retrospective series, and a limited number of observational studies. Therefore, these observations should be interpreted with caution, and definitive conclusions regarding prognosis cannot yet be drawn.

Pulmonary involvement appears to be another area of divergence between classic and overlap cases. In traditional anti-GBM disease, diffuse alveolar haemorrhage is a well-known feature. However, data from larger series and pooled reviews suggest that pulmonary bleeding is relatively uncommon in the overlap population [5,6,9]. This pattern has led to the perception that the overlap form tends to follow a more kidney-limited or indolent course. Nonetheless, the case presented here, marked by severe pulmonary haemorrhage, demonstrates that life-threatening pulmonary-renal involvement can still occur. The presence of mesangial IgA deposits does not rule out active and severe anti-GBM autoimmunity. Other atypical or severe presentations have also been reported, including patients with stable renal function but strong immunological activity [7,11].

Renal biopsy remains critical for confirming the diagnosis of this overlap entity. The defining feature is the combination of strong, diffuse linear IgG staining along the glomerular basement membranes with granular, IgA-dominant, mainly mesangial immune complex deposition [5,6,9,12]. An important diagnostic consideration in such cases is distinguishing true concurrent IgAN from incidental mesangial IgA deposition, which may occasionally be observed in other glomerular diseases and does not necessarily indicate clinically significant IgAN. Accordingly, immunofluorescence findings must be interpreted in conjunction with light microscopic features and, where available, electron microscopy. In the present case, the diagnosis of concurrent IgAN was supported by the concordance of the light microscopic, immunofluorescence, and electron microscopic findings. Light microscopy demonstrated mesangial hypercellularity with segmental sclerosis, immunofluorescence showed dominant mesangial IgA deposition accompanied by C3, and electron microscopy confirmed mesangial and paramesangial electron-dense immune-type deposits. Taken together, these concordant morphologic, immunopathological, and ultrastructural findings support the diagnosis of concurrent IgAN rather than incidental mesangial IgA deposition or nonspecific mesangial IgA trapping [16,17].

The mechanism underlying this overlap is still not fully understood. One widely discussed theory is the “IgA-first” hypothesis, which proposes that pre-existing or subclinical IgAN causes chronic injury to the mesangium and glomerular basement membrane. This damage may unmask hidden epitopes within the α3(IV) collagen chain, triggering an autoimmune response and the development of anti-GBM antibodies [9,18]. Several case reports describe patients with longstanding haematuria or proteinuria before the onset of anti-GBM disease. Additionally, many overlap cases have been reported in regions where IgAN is highly prevalent [5,6,9,19]. Still, this theory remains speculative. Other explanations, such as common environmental exposures, infection-driven immune activation, or shared autoimmune predisposition, remain plausible [1,9,12,13]. Multiple pathways may contribute to the development of this overlap phenotype.

Therapeutically, the consensus is to treat based on the dominant anti-GBM component during the acute phase. Plasma exchange, high-dose corticosteroids, and cyclophosphamide remain the standard of care when anti-GBM antibodies are present, especially in patients with pulmonary haemorrhage or rapidly worsening kidney function [12,13]. The concurrent presence of IgAN should not delay or limit this treatment approach. In this case, early use of plasma exchange and immunosuppressive therapy, along with bronchial artery embolisation to manage bleeding, led to rapid improvement in respiratory status, similar to what has been reported in other recent overlap cases [7,19].

Emerging reports have also highlighted the possible role of rituximab in complex or treatment-resistant cases. For example, Bandak et al. documented successful use of rituximab in a patient with anti-GBM disease coexisting with membranous nephropathy, offering potential insight into alternative treatment strategies [20]. Although such reports are still limited, they suggest that B-cell-targeted therapies may be worth considering in selected situations where conventional treatments are not suitable.

Despite more optimistic findings in the overlap group, long-term outcomes still depend on familiar prognostic markers, namely baseline kidney function, need for dialysis at presentation, and the extent of chronic and crescentic injury seen on biopsy [5,6,9]. These factors remain relevant even in this subgroup. Additionally, persistent urinary abnormalities related to IgAN may complicate follow-up and require careful evaluation after the anti-GBM component has been addressed.

## Conclusions

In conclusion, the overlap of anti-GBM disease and IgAN represents a rare but increasingly recognised clinicopathological entity with a clinical course that often differs from conventional anti-GBM disease. While many cases follow a more favourable renal trajectory, severe pulmonary-renal involvement is still possible. Accurate diagnosis depends on thorough clinicopathological assessment. In the acute setting, treatment should be directed toward anti-GBM activity, while long-term management must take into account residual IgA-mediated disease and known prognostic factors.
